# Validation of the hospital frailty risk score in China

**DOI:** 10.1007/s41999-025-01212-0

**Published:** 2025-05-02

**Authors:** Yue Qiu, Weiqing Xiong, Xinyue Fang, Pei Li, Simon Conroy, Laia Maynou, Kenneth Rockwood, Xien Liu, Ji Wu, Andrew Street

**Affiliations:** 1 Tsinghua Medicine, Tsinghua University, Haidian District, Beijing, 100084 China; 2 Tsinghua Medicine, Tsinghua University, Haidian District, Beijing, 100084 China; 3 Tsinghua Shenzhen International Graduate School, Tsinghua University, Shenzhen, Guangdong 518055 China; 4 Wolfson Institute of Population Health, Queen Mary University of London, Mile End Road, E1 4NS London, UK; 5 Department of Econometrics, Statistics and Applied Economics, Universitat de Barcelona, Barcelona, Spain; 6 Division of Geriatric Medicine, Frailty Elder Care Network, Nova Scotia Health, Dalhousie University, Halifax, NS B3H2E1 Canada; 7 Department of Electronic Engineering, Tsinghua University, Beijing, 100084 China; 8 College of AI, Tsinghua University, Beijing, 100084 China; 9 Department of Health Policy, London School of Economics and Political Science, Houghton Street, London, WC2A 2AE UK

**Keywords:** Hospital frailty risk score, HFRS, Frailty risk assessment, Length of stay, Hospital costs

## Abstract

**Purpose:**

To validate the Hospital Frailty Risk Score (HFRS) in Chinese hospital settings, describing how patients are allocated to frailty risk groups and how frailty risk is associated with length of stay (LoS) and hospital costs.

**Design:**

Retrospective observational study.

**Setting:**

Forty-eight hospitals in Lvliang City, Shanxi Province, China.

**Subjects:**

Patients aged 75 years or older hospitalised between 1 January 2022 and 31 December 2023 (n = 34,731).

**Methods:**

A logistic regression model examined the association between long length of stay (LoS) and frailty risk. A generalised linear model assessed the association between hospital costs and frailty risk. Subgroup analyses of age group, sex, and hospital tiers were conducted.

**Results:**

22.2% of patients were categorised as having zero risk, 62.4% as low risk, 15.3% as intermediate risk, and 0.08% as high risk. Compared to the zero risk group: for those with low risk, the probability of long LoS was 1.92 (95% CI 1.79–2.06) times higher and hospital costs were ¥1926 (95% CI 1655–2197) higher; for those with intermediate risk, the probability of long LoS was 2.7 (95% CI 2.49–2.96) times higher and hospital costs were ¥4284 (95% CI 3916–4653) higher; and for those with high risk, the probability of long LoS was 6.7 (95% CI 3.06–14.43) times higher and hospital costs were ¥16,613 (95% CI 12,827–20,399) higher. The explanatory power of the HFRS held across subgroups.

**Conclusions:**

Compared to patients aged 75 + elsewhere, those in China had lower frailty risk scores, likely reflecting a younger age structure and recording of fewer diagnosis codes. Even so, the HFRS is a powerful predictor of long length of stay and hospital costs in China.

**Supplementary Information:**

The online version contains supplementary material available at 10.1007/s41999-025-01212-0.

## Key summary points

**Aim:**

To validate the Hospital Frailty Risk Score (HFRS) in Chinese hospital settings

**Findings:**

Relatively few older people hospitalised in China are categorised using the HFRS as having high frailty risk.

The HFRS predicts long length of stay and hospital costs among hospitalised older patients in China.

**Message:**

The HFRS has potential for widespread use in both developed and developing countries that use ICD-10 codes.

**Supplementary Information:**

The online version contains supplementary material available at 10.1007/s41999-025-01212-0.

## Introduction

Health issues among older people are a significant concern in ageing societies, impacting individual quality of life and the sustainability of healthcare systems. Frailty is defined as an “age-related, clinically identifiable state of diminished physiologic reserve and increased vulnerability to a broad range of adverse health outcomes” [1]. The associations between frailty and length of stay, hospital costs, and mortality have been confirmed in many studies [2]. Frailty significantly contributes to functional deterioration in older people [3], increasing healthcare demands and burdening families and society.

Global recognition of the need to assess and manage frailty has led to the development of various assessment tools [4–11]. By identifying frailty-related risks, particularly in the first hours of an acute admission [12–15], it may be possible to mitigate harms such as reduced mobility, which is associated with death or institutionalisation [16], loss of lean muscle mass, pressure ulcer development [17], incontinence, thrombosis, constipation, pain, infections, and low mood [18]. However, many tools are too complex for use in acute care settings [19] and require professional evaluations, complicating their integration into clinical care [20, 21]. To address this, Gilbert and colleagues developed the Hospital Frailty Risk Score (HFRS), which uses International Statistical Classification of Diseases and Related Health Problems, tenth revision (ICD-10) codes from electronic health records for hospitalised patients to compute frailty risk scores without additional data gathering [22].

Since its development, the HFRS has garnered extensive attention, with applications to different types of patients to validate its predictive capacity for outcomes, such as length of stay, mortality, and treatment costs [23–31]. These validation studies, however, have largely been conducted in developed countries, with few applications from developing ones. To start addressing this gap, this paper applies the HFRS to older patients admitted to hospital in China.

Several studies have measured the prevalence of frailty in the Chinese community, using instruments such as the Frailty Index [32] and a validated physical frailty phenotype (PFP) scale [33]. A meta-analysis showed that 15% of Chinese community-dwelling older people aged 75–84 experienced frailty, while this figure rose to 25% for those aged 85 and over [34]. As a rapidly ageing developing country, there is a great need to identify and manage frailty in older people in China. However, China has few healthcare workers relative to the size of the population (2.4 physicians and 3.3 nurses and midwives per 1000 [35]), which rules out widespread application of many frailty assessment tools that require primary data collection. Using electronic health records to identify frail patients offers a more cost-effective approach in China.

This study aimed to assess the applicability of the HFRS for hospitalised patients 75 years and older in China, with a focal emphasis on the effectiveness of frailty identification and the tool’s ability to predict healthcare resource use. The findings will provide evidence on whether China can adopt the HFRS or if a new tool needs to be developed based on China’s specific conditions.

## Methods

### Setting

Health care in China combines a mix of public and privately funded care [36]. Over 95% of the population is insured through either the Urban Employee Basic Medical Insurance (UEBMI) scheme, which provides coverage for urban employees, or through the Urban and Rural Residence Basic Medical Insurance (URRBMI) scheme which covers those without formal employment in urban and rural settings. These insurance schemes offer similar benefits and together account for 46% of total health expenditure, with 26.7% coming from government sources and 27.3% from individuals, mainly as out-of-pocket payments [37].

We conducted a retrospective study of patients aged 75 and older who had been hospitalised between 1 January 2022 and 31 December 2023 in Lvliang City, Shanxi Province, China. Lvliang City has a middle-ranged economic level among the cities of China and, according to the 7th China population census [38], has a permanent population of about 3.3 million, of which 3.9% are aged 75 years and above, and 0.8% are aged 85 years and above. It is a relatively young city as those proportions for the whole country are 4.8% and 1.1%, respectively [38].

### Data

We used a medical database to retrieve anonymised patient-level data. The entire database contained routine administrative medical data, including clinical characteristics and healthcare utilisation, for 357,186 patients admitted to 72 primary hospitals, 45 secondary hospitals, and 3 tertiary hospitals in Lvliang City from 1 January 2022 to 31 December 2023. In the hospital classification of China, primary hospitals are community facilities with fewer than 100 beds that provide preventive care, primary care, and rehabilitation. Secondary hospitals have between 100 and 500 beds, are located in cities, and offer broad services and some specialities. Tertiary hospitals are located in major cities and offer a wide range of specialists and have more than 500 beds. In tertiary hospitals, staff tend to be more qualified and equipment more advanced, so complexity and quality should be higher in these hospitals [39].

**Fig. 1:**
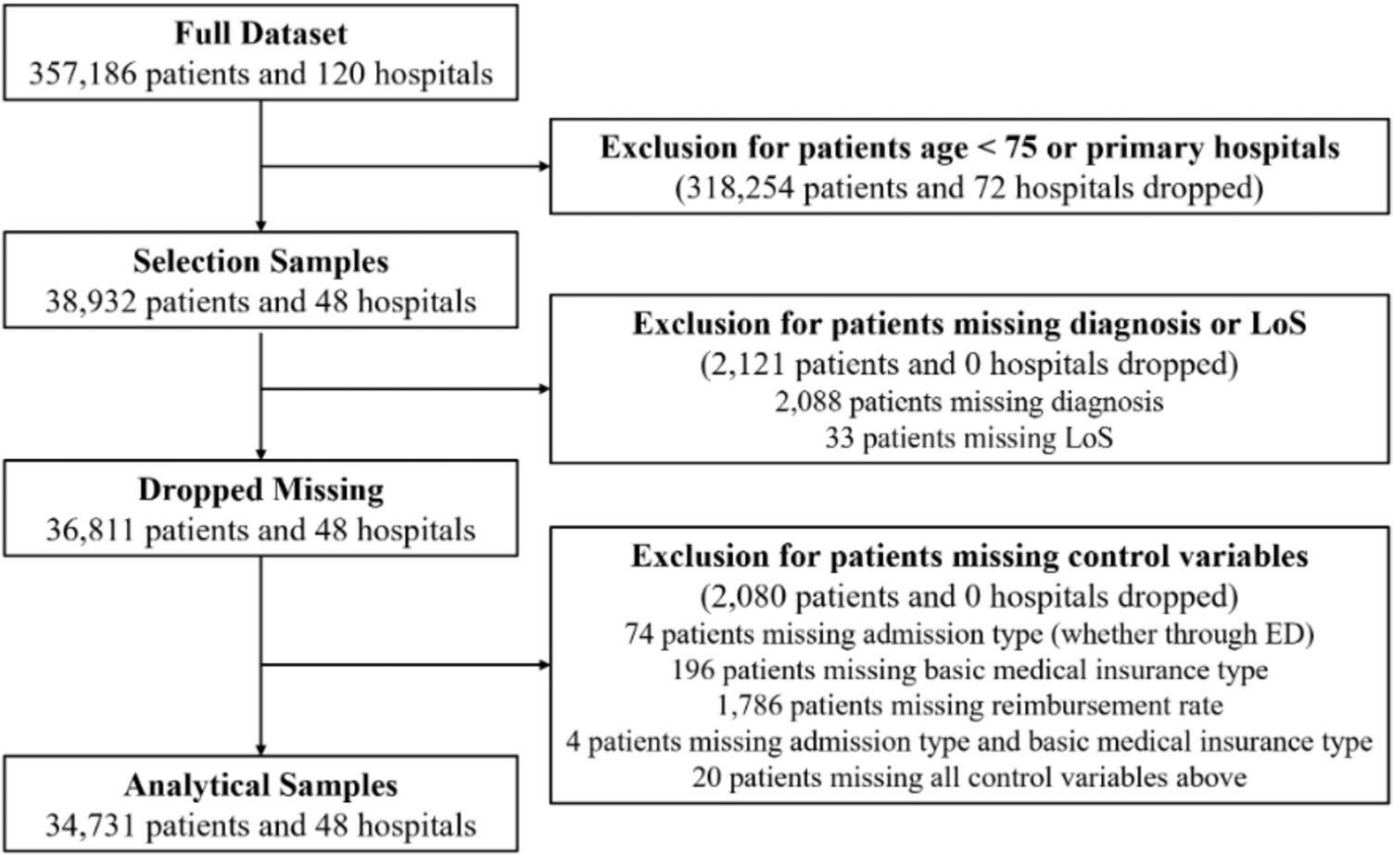
Flow diagram of sample selection

The process of selecting the analytical sample is described in Fig. 1. We extracted information for all 38,932 patients aged 75 years and over admitted to secondary and tertiary hospitals, dropping patients admitted to primary hospitals for consistency with Gilbert et al. [22]. For patients with multiple admissions, only the most recent admission was used for the analysis. After excluding 4201 (10.8%) patients with missing data, the analytical sample comprised 34,731 patients. We compared key variables before and after excluding patients with missing data for other variables, reported in Appendix A1. This revealed no significant differences except for hospital costs.

### Outcome and frailty measures

We analysed the relationship between the HFRS and two outcomes, long LoS and hospital costs, controlling for other characteristics:

- We used the patient’s admission and discharge dates to calculate LoS. Consistent with the original HFRS study by Gilbert et al. [22], this was converted into a binary variable where 1 indicated LoS over 10 days, 0 otherwise, and analysed using a logistic regression model. As a robustness check, we also analysed LoS using a Poisson model, reported in Appendix A5.
- The cost of the hospital stay is recorded in the patient’s medical record, this being the sum of the patient’s treatment costs for the admission, including the cost of examinations, tests, medicines, consumables, surgical treatment, and medical services. Costs were analysed using a generalised linear model.

For each patient, the HFRS was calculated by combining a weighted set of 109 3-character ICD-10 diagnostic codes [22]. Given that only 2 year's worth of data were available, we constructed the *HFRS*(*a*) form, using diagnostic information from the current admission only [30]. The HFRS takes values from 0 to 173.2. Commonly patients have been categorised as having low (HFRS < 5), intermediate ((5 ≤ HFRS < 15), or high (HFRS ≥ 15) frailty risk [22]. However, in this study, a large proportion of patients had an HFRS of zero and, hence, we categorised patients into four groups: zero frailty risk (HFRS = 0), low frailty risk (0 < HFRS < 5), intermediate frailty risk (5 ≤ HFRS < 15), or high frailty risk (HFRS ≥ 15). Those in the zero frailty risk category formed the reference group in the regressions.

The analyses controlled for patient age, sex, Charlson comorbidity index (CCI) [40–43], admission via the emergency department, the number of operations performed, hospital type, and the patient’s insurance scheme. The Appendix details the construction of these control variables and provides specification details about the regression models.

### Subgroup analyses

We conducted subgroup analyses to uncover the relationship between frailty risk and patient outcomes among patients with different characteristics. The subgroups were divided according to age (75–79, 80–84, 85 +), sex (male and female), and hospital tiers (secondary and tertiary). The regression models and the control variables in the subgroup analyses were the same as those employed in the analysis of all patients, only excluding the variable used to form the subgroup.

## Results

### Descriptive statistics

The analytical sample comprised 34,731 patients aged 75 and above. Descriptive statistics are shown in Table 1. Among these patients, the maximum HFRS score was 19.8 points, and 7715 (22.2%) patients were categorised as having zero frailty risk, 21,667 (62.4%) as low frailty risk, 5320 (15.3%) as intermediate frailty risk, and only 29 (0.08%) as high frailty risk. Generally, patients with higher frailty risk were more likely to stay in hospital for more than 10 days, had higher costs, were more likely to be admitted through the ED, and had a higher CCI score.

**Table 1:** Descriptive statistics

Variables			Descriptive statistics (N = 34,731)					
	Zero risk (n = 7715, 22.21%)		Low risk (n = 21,667, 62.39%)		Intermediate risk (n = 5320, 15.32%)		High risk (n = 29, 0.08%)	
	N/Mean	Proportion/SD	N/Mean	Proportion/SD	N/Mean	Proportion/SD	N/Mean	Proportion/SD
*Long length of stay*								
Yes	1178	15.27%	5443	25.12%	1832	34.44%	18	62.07%
No	6537	84.73%	16,224	74.88%	3488	65.56%	11	37.93%
*Hospital costs ¥*								
	5931	6104	7387	8766	11,596	21,251	28,324	48,904
*Age group*								
75–79	4099	53.13%	10,754	49.63%	2240	42.11%	14	48.28%
80–84	2326	30.15%	6796	31.37%	1821	34.23%	10	34.48%
85 +	1290	16.72%	4117	19.00%	1259	23.67%	5	17.24%
*Sex*								
Male	3941	51.08%	10,839	50.03%	2704	50.83%	15	51.72%
Female	3774	48.92%	10,828	49.97%	2616	49.17%	14	48.28%
*Hospital tier*								
Secondary	6075	78.74%	15,843	73.12%	2983	56.07%	11	37.93%
Tertiary	1640	21.26%	5824	26.88%	2337	43.93%	18	62.07%
*Admission through ED*								
Yes	531	6.88%	2076	9.58%	772	14.51%	7	24.14%
No	7184	93.12%	19,591	90.42%	4548	85.49%	22	75.86%
*Charlson comorbidity index (CCI)*								
0	6861	88.93%	18,231	84.14%	4195	78.85%	16	55.17%
1	772	10.01%	3060	14.12%	973	18.29%	10	34.48%
2 +	82	1.06%	376	1.74%	152	2.86%	3	10.34%
*Number of operations*								
	0.429	0.707	0.283	0.736	0.526	1.102	1.207	2.007
*Basic medical insurance type*								
URBMI	6262	81.17%	17,396	80.29%	4117	77.39%	19	65.52%
UEBMI	1453	18.83%	4271	19.71%	1203	22.61%	10	34.48%
*Reimbursement rate*								
	0.69	0.162	0.686	0.159	0.672	0.166	0.722	0.153

### Regression results

**Fig. 2:**
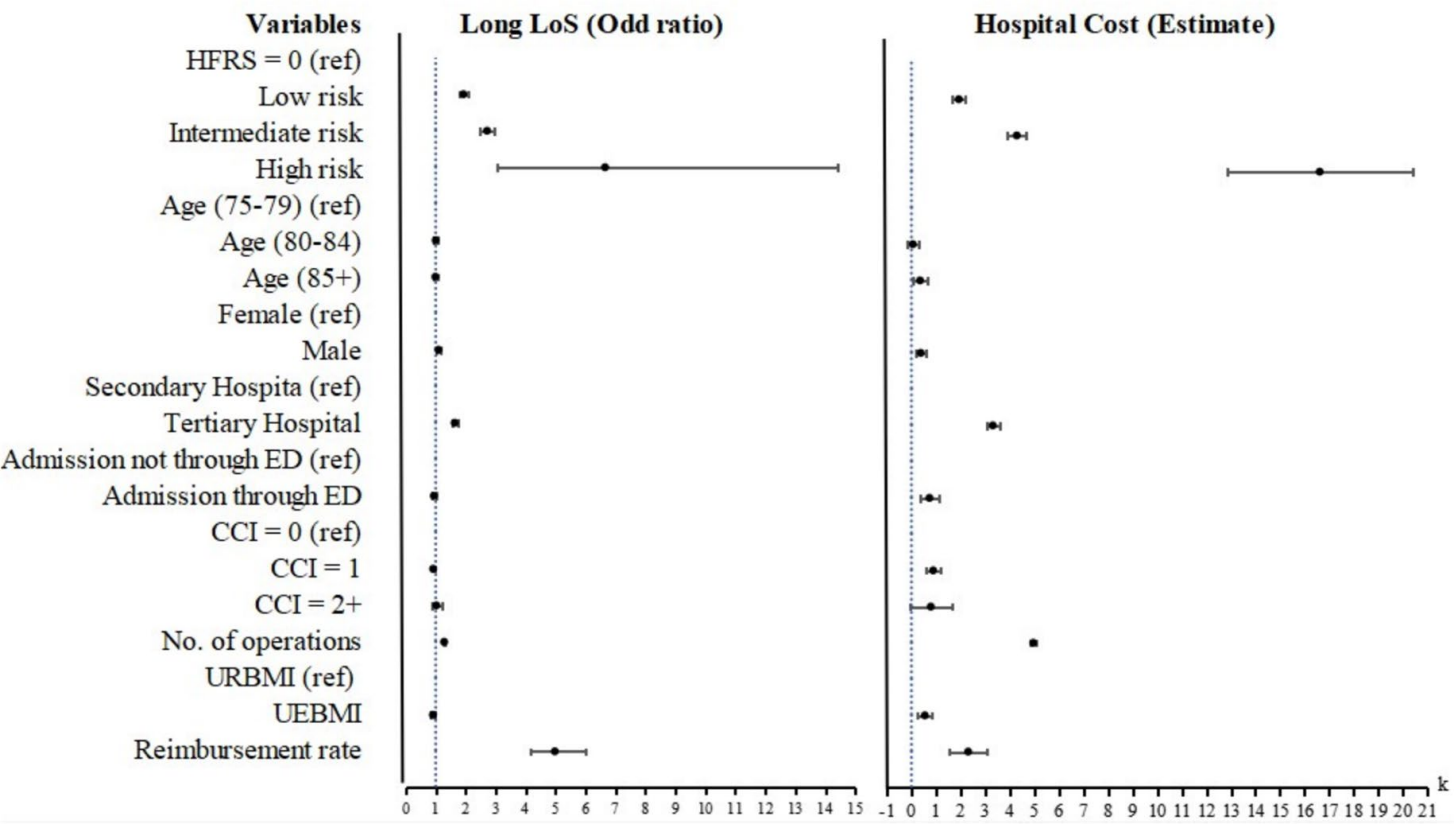
Forest plot of regression results for the full analytical sample

The full regression results for the analyses of long LoS and hospital cost are reported in Appendix Table A2 and summarised as a forest plot in Fig. 2. Compared to the zero frailty risk group, the probability of long LoS was: 1.92 [95% confidence interval (CI) 1.79–2.06] times higher for those with low frailty risk; 2.71 (95% CI 2.49–2.96) times higher for those with intermediate frailty risk; and 6.65 (95% CI 3.06–14.43) times higher for those with high frailty risk. The wide CI for the high frailty group reflects the small number of patients in this group. The results of applying the Poisson model to analyse LoS are consistent with the logistic model, as reported in Appendix A5.

Hospital costs also increased in line with the frailty risk level. Compared to those with zero frailty risk, costs were ¥1926 (95% CI 1655–2197) higher for those with low frailty risk, ¥4284 (95%CI 3916–4653) higher for those with intermediate frailty risk, and ¥16,613 (95% CI 12,827–20,399) higher for those with high frailty risk.

Most control variables revealed significant effects for long LoS or hospital costs. For instance, LoS and hospital costs increased with the number of operations (long LoS OR 1.28 95% CI 1.24–1.32; hospital costs ¥4955 95% CI 4817–5093). In the analysis of long LoS, the odd ratios for CCI were significant but less than 1 (OR 0.91 95% CI 0.85–0.98) for CCI = 1 and insignificant for CCI = 2 + . For hospital costs, the CCI coefficients were positive (CCI = 1 ¥882 95% CI 564–1202; CCI = 2 + ¥786 95% CI − 49–1622).

### Subgroup analyses

The regression results for length of stay and hospital costs across subgroups are summarised in Appendix A6.

Across all subgroups, compared to those with zero frailty risk, both LoS and costs increased across the frailty risk categories. For those with low frailty risk, the differences were small; for those in the intermediate frailty risk category, the differences are larger and significant; for those in the high frailty risk category, there are wide confidence intervals around the point estimates because of the small numbers in this category.

These patterns of longer LoS and higher costs as frailty risk increases demonstrate that the HFRS can be applied across age categories, sex, and hospital tiers, thereby underscoring its usefulness as a predictive tool for patients hospitalised in China.

## Discussion

This paper had two objectives. First, to validate the HFRS in Chinese hospital settings population, describing how patients are allocated to frailty risk groups. Second to assess how frailty risk is associated with LoS and hospital costs.

As regards the first objective, in the Lvliang hospital care setting, 7715 (22.2%) individuals were identified as having zero frailty risk, 21,667 (62.4%) patients were categorised to the low frailty risk group, 5320 (15.3%) into the intermediate frailty risk group, and 29 (0.08%) into the high frailty risk group. Notably, very few were identified as being high risk, especially when compared with much higher proportions in the high-risk group in two studies from England (20% in [22] and in 21.8% [30]) and of 17% in a study from France [29]. However, other studies of those over 75 years also report small proportions in the high-risk group: 2.9% in a study from Switzerland [24], 2.6% in one from Canada [23], and 1.9% in a study from Australia [44].

**Fig. 3:**
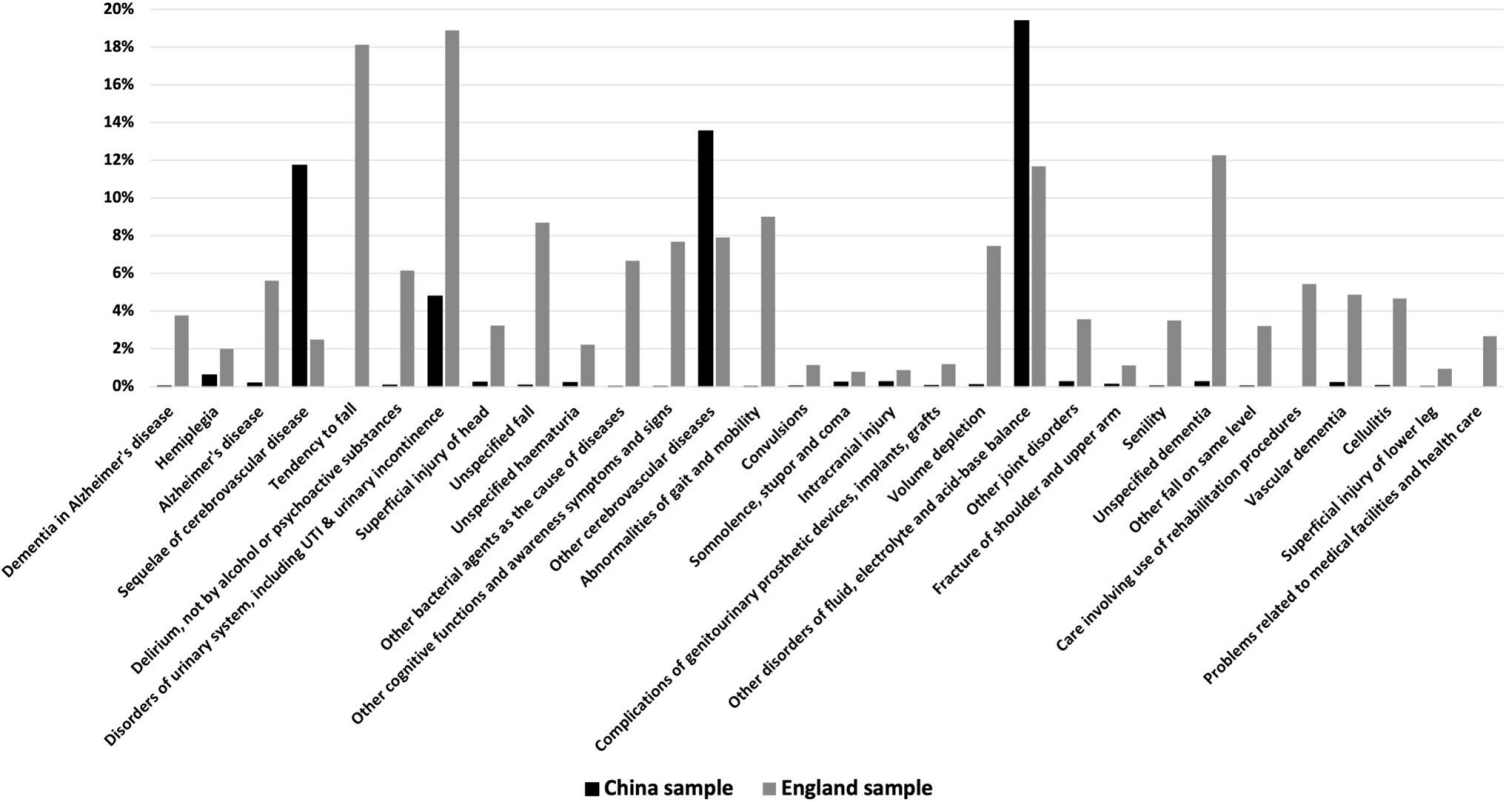
Proportion of sample with each ICD-10 diagnosis

The HFRS for any particular individual is driven by whether they have one of the 109 ICD-10 codes and the weight attached to that code, these weights ranging from 0.1 to 7.1 (F00 Dementia in Alzheimer’s disease). The proportions of the sample with the 30 ICD-10 codes with a weight > 2.0 are shown in Fig. 3 and Table 2. These proportions are compared to patients aged 75 and above hospitalised in England between 2013 and 2019 [45].

**Table 2:** Distribution of ICD-10 codes with HFRS weight > 2.0

ICD-10 code	ICD-10 description	Weight	China sample (%)	England sample (%)
F00	Dementia in Alzheimer’s disease	7.1	0.04	3.77
G81	Hemiplegia	4.4	0.61	2.01
G30	Alzheimer’s disease	4.0	0.20	5.62
I69	Sequelae of cerebrovascular disease	3.7	11.75	2.50
R29	Tendency to fall	3.6	0.00	18.13
F05	Delirium, not by alcohol or psychoactive substances	3.2	0.07	6.15
N39	Disorders of urinary system, including UTI & urinary incontinence	3.2	4.79	18.89
S00	Superficial injury of head	3.2	0.23	3.23
W19	Unspecified fall	3.2	0.07	8.69
R31	Unspecified haematuria	3.0	0.20	2.22
B96	Other bacterial agents as the cause of diseases	2.9	0.01	6.68
R41	Other cognitive functions and awareness symptoms and signs	2.7	0.01	7.70
I67	Other cerebrovascular diseases	2.6	13.55	7.91
R26	Abnormalities of gait and mobility	2.6	0.01	9.01
R56	Convulsions	2.6	0.04	1.15
R40	Somnolence, stupor and coma	2.5	0.23	0.80
S06	Intracranial injury	2.4	0.25	0.88
T83	Complications of genitourinary prosthetic devices, implants, grafts	2.4	0.05	1.19
E86	Volume depletion	2.3	0.10	7.47
E87	Other disorders of fluid, electrolyte and acid–base balance	2.3	19.40	11.69
M25	Other joint disorders	2.3	0.26	3.58
S42	Fracture of shoulder and upper arm	2.3	0.13	1.13
R54	Senility	2.2	0.04	3.50
F03	Unspecified dementia	2.1	0.27	12.27
W18	Other fall on same level	2.1	0.02	3.21
Z50	Care involving use of rehabilitation procedures	2.1	0.00	5.44
F01	Vascular dementia	2.0	0.20	4.87
L03	Cellulitis	2.0	0.05	4.66
S80	Superficial injury of lower leg	2.0	0.01	0.95
Z75	Problems related to medical facilities and healthcare	2.0	0.00	2.66

Clear differences are evident. Less than 1% of the Lvliang sample had one of the three ICD-10 codes (F00, G81, and G30) with the highest weight; in England, 11.4% of the sample had one of these codes. In the Lvliang dataset, the highest proportion is attributed to the ICD-10 code for “E87 Other disorders of fluid, electrolyte and acid–base balance” (19.4%), followed by “I67 Other cerebrovascular disease” (13.6%) and “I69 Sequelae of cerebrovascular disease” (11.8%). In the English dataset, the distribution of the 30 ICD-10 codes is more spread out, with the highest proportions attributed to “N39 Disorders of urinary system including UTI & urinary incontinence” (18.9%) and “R29 Tendency to fall” (18.1%). Comparing these two samples, it is evident that different ICD-10 codes identify the frailty risk of patients in the two countries.

The most direct reason for the differences between the Chinese and English data is the lower count of the 109 ICD-10 codes used to construct the HFRS in the Lvliang dataset, particularly for codes with large HFRS weights such as Dementia (F00) [46] and Alzheimer’s disease (G30) [47]. The lower frequency of these codes in China can be attributed partly to the younger age structure and its associated disease spectrum. Compared to 42.5% of the population aged 85 years or older reported in a regional study in England [30], Lvliang’s patients are relatively young, with only 19.2% of the population aged 85 years or older. Due to this younger age structure, diseases like dementia and Alzheimer’s, which are more prevalent in older age groups, have lower incidence rates in Lvliang. Consequently, the frequency of these ICD-10 codes is also lower, resulting in lower risk scores when calculating the HFRS. Besides, the limited availability of well-trained medical records staff might mean that diagnoses are under-coded in China [48, 49].

Disparities in healthcare delivery capacity between countries and regions may also play a role in explaining the differences in the proportions of patients in each frailty risk group [50]. Around 2.5% of patients in China seek care outside the region in which they live [51], the primary reason being to access higher quality care [52]. Patients from Lvliang might seek high-quality healthcare in nearby larger cities like Datong or Beijing [53]. These factors will mean that hospitals will be treating quite different patient profiles, implying that the HFRS is influenced by factors other than frailty-related diagnostic codes [24] and underscoring a need for further investigation into the underlying causes as well as possible coding differences [44]. Note that, as well as impacting the HFRS, coding differences also have implications for the CCI, where the proportion of patients with a CCI ≥ 2 was 1.76% in our study but 51.6% in the study in the England [30]. Low CCI scores were also reported in a study of older patients admitted to hospital in Beijing [54].

Regarding the second objective, despite differences in patient profiles and diagnostic coding, the HFRS still emerged as a strong explanator or length of stay and costs for patients admitted to hospital in Lvliang city. Patients in low, intermediate, and high HFRS risk groups were significantly and progressively more likely to stay in hospital for over 10 days and have higher treatment costs compared to patients in the zero risk group. If diagnostic coding were improved, frailty risk scores would be higher and the explanatory power of the HFRS would become greater. Even so, the HFRS had greater explanatory power for these two outcomes than any of the other variables included in the regression analyses. Subgroup analyses further confirmed the robustness of these findings.

For long LoS, our regression results were similar to other HFRS validation studies conducted in different countries [22–24, 28, 30, 39, 55, 56]. The influence of the HFRS on hospital costs was also consistent with findings from other studies [24, 28]. Our validation study suggests that, despite variations in healthcare system and ICD-10 coding rules across countries, the risk of frailty calculated using the HFRS methodology is a useful predictor of length of stay and hospital costs in China, as has been found elsewhere [57]. Therefore, the HFRS holds great potential for widespread use in countries using ICD-10 codes, both in developed and developing countries, due to its explanatory power, convenience, and cost-effectiveness.

There are limitations to this study. First, the narrow data window only allowed analysis of the patient’s most recent admission, which will have led to a lower HFRS compared to other studies that also include diagnostic information from the previous two admissions within the last two years, as recommended [30]. This means that, on the one hand, diagnoses recorded in a patient’s previous admissions are not captured, but, on the other hand, the HFRS is constructed in a consistent fashion for every patient in the study. Second, diagnoses may have been under-coded. If so, the explanatory power of the HFRS would have been under-estimated. Third, a high proportion of patients were omitted from analysis due to missing data. With the exception of costs, data appeared to be missing at random, thereby suggesting that the analytical sample remained representative. Nevertheless, the high proportion highlights the scope for improved coding practice, not just of diagnoses but more generally. Fourth, we did not analyse the relationship between the HFRS and in-hospital death because, due to Chinese cultural practices, most older patients prefer to receive end-of-life care at home with family and friends [58]. Reflecting this, only 0.37% died in hospital. Nor did the data allow us to identify those who were discharged to die at home. Fifth, this study utilised regional data from a single city in China, which may not be representative of the entire country, though it may be fairly typical of other middle-ranged cities with similar socio-economic characteristics. Future studies of the HFRS using data from other areas in China would be welcome.

## Conclusions

As the population ages, particularly in low-income and middle-income countries, the impact of frailty will escalate [2]. Frailty risk, easily calculated using the HFRS, offers benefits at the micro-, meso-, and macro-level of the system. At the micro-level, involving clinician–patient interaction, a measure of frailty risk alerts the clinician to the potential prognosis. People with high HFRS scores have an increased risk of dying in hospital—this might prompt the clinician to activate critical care if clinically appropriate and in keeping with the patient’s wishes and preferences. Alternatively, it might lead to a more palliative or supportive paradigm of care being instituted, following assessment of the individual. Fundamentally assessment of frailty risk moves us away from a one-size-fits-all approach, recognising that patients are at different stages on their life trajectory.

At the meso-level, it allows hospitals to match resources to patient needs. For example, it might be that people with high HFRS scores are found in all parts of the hospital, prompting the development of a geriatric liaison service and evaluating its impact on the available service metrics.

At the macro-level, it facilitates the creation of registries, which can track the flow of a risk stratified cohort of older people along care pathways and redirect where appropriate. For example, a dynamic frailty risk registry might highlight that a patient with high frailty discharged from hospital has not been referred to community services. This can then be rectified by putting post-discharge support in place.

Our study is the first to confirm the predictive effect of HFRS on length of stay and hospital costs in China, a developing country with a growing older population. The HFRS strikes a balance between broad applicability and low cost through its big data-driven approach. However, the identification of frailty risk by HFRS in this study was significantly different from the original English population cohorts by Gilbert et al. [22], likely due to the differences in age structure, disease spectrum, healthcare delivery capacity, and diagnostic coding practices between England and China. In light of these findings, developing countries like China might benefit from employing the HFRS but also from using a similar big data-driven approach to develop localised frailty screening tools, tailored to reflect their particular the demographic and healthcare landscapes [59]. Investment in recruitment and training of coding staff should improve diagnostic coding practices and data quality. In places with the necessary infrastructure, pilot implementation of HFRS in clinical workflows could be a good way to inform how construction of the HFRS might be refined and how it might best be applied to assess frailty risk among hospitalised patients in other developing countries.

## Supplementary Information

Below is the link to the electronic supplementary material.

Supplementary file1 (PDF 1501 KB)

## Data Availability

Chinese data presented in this study may be obtained from a third party on request from the authors and are not publicly available. The English data came from the Hospital Episode Statistics provided by NHS Digital under Data Sharing Agreement NIC-354497-V2J9P. These data may be obtained from a third party and are not publicly available. This paper has been screened to ensure that no confidential information is revealed.
