# Supplementary material for: Validation of the hospital frailty risk score in China
Source: Eur Geriatr Med. 2025 May 2;16(4):1319–28. doi: 10.1007/s41999-025-01212-0 (PMC12378284; doi:10.1007/s41999-025-01212-0)
Supplement: Supplementary file 1 — Supplementary file1 (PDF 1501 KB) [file 41999_2025_1212_MOESM1_ESM.pdf]

Validation of the Hospital Frailty Risk Score in China

Appendix

Yue Qiu, Weiqing Xiong, Xinyue Fang, Pei Li, Simon Conroy, Laia Maynou, Kenneth Rockwood, Xien Liu, Ji Wu, Andrew Street

A Appendix

A.1 Missing data

As reported in Figure 1 in the main paper, 4,201 (10.8%) of the selection sample were dropped from analysis due to missing data relating to either outcome or control variables. We used Pearson's chi-squared test to compare categorical variables before and after excluding patients with missing data for other variables. The results were reported in Table A1. No significant differences were found in long LoS, HFRS group, age, sex, and CCI. For hospital costs we conducted a t-test on log transformed costs (to correct for the skewed distribution). Even though mean costs are little different between the before and after samples, this difference proved significant, unsurprisingly given the large sample size.

Table A1: Data structure before and after exclusion

**Variables**	**Before (N=38,932)**		**After (N=34,731)**		**Test results**	
	N/Mean	Proportion/SD	N/Mean	Proportion/SD	χ^2/t value	p value
**Long length of stay**						
Yes	9,527	24.47%	8,471	24.39%	0.102	0.749
No	29,372	75.44%	26,260	75.61%		
**Hospital costs**						
Natural	7,980	11,683	7,726	11,434		
Log	8.64	0.771	8.63	0.737	-3.144	0.002
**HFRS group**						
Zero	8,196	21.05%	7,715	22.21%	0.069	0.995
Low	22,998	59.07%	21,667	62.39%		
Intermediate	5,620	14.44%	5,320	15.32%		
High	30	0.08%	29	0.08%		
**Age group**						
75-79	19,321	49.40%	17,107	49.26%	0.66	0.719
80-84	12,285	31.56%	10,953	31.53%		
85+	7,416	19.05%	6,671	19.21%		
**Sex**						
Male	19,460	49.98%	17,499	50.38%	1.174	0.279
Female	19,472	50.02%	17,232	49.62%		
**Charlson Comorbidity Index (CCI)**						
0	31,017	79.67%	29,303	84.37%	0.473	0.789
1	5,171	13.28%	4,815	13.86%		
2+	656	1.68%	613	1.77%		

A.2 Control variables

The analyses controlled for patient age, categorised into three age bands (75-79, 80-85, and 85+, the former forming the reference group), and biological sex (female being the reference category).

Clinical complexity was captured using the Charlson comorbidity index (CCI) [1; 2; 3; 4], which uses age and ICD-10 comorbidity indicators to estimate mortality risk. The CCI takes values from 0 to 17 but was categorised for the analysis into three groups (0,1, and 2+), with the first group forming the reference category. Other studies have demonstrated that the HFRS has significantly explanatory power over and above the CCI (eg [5]). Whilst there are some similarities between the CCI and the HFRS, the HFRS uses a wider range of ICD-10 codes, including frailty related syndromes such as falls, and more items on cognition. This likely contributes to the added discrimination that the HFRS confers over and above the CCI.

We accounted for whether the patient was admitted via the emergency department (ED) and the number of operations performed.

We took account of the type of hospital to which the patient was admitted to capture unobserved casemix differences between hospital tiers, with secondary hospitals forming the reference category. As a proxy measure for socio-economic status we also accounted for whether the patient was insured under either the UEBMI, in which case they were in formal employment, or the URBMI scheme, which covers everyone else, these forming the reference group. We also calculated the reimbursement rate as payments by medical insurance divided by total hospital costs, to account for the percentage of medical insurance reimbursement.

A.3 Estimation model

We analysed the relationship between the HFRS and the two outcomes, long LoS and hospital costs, controlling for other characteristics. The regression models took the general form below, recognising that patients $i = 1...I$ are clustered in hospitals $j = 1...J$:

$$y_{ij} = \alpha + \delta_L HFRS_{ij}^L + \delta_I HFRS_{ij}^I + \delta_H HFRS_{ij}^H + \sum_{m=1}^{M} \beta_m X_{ij} + u_j + \epsilon_{ij} \qquad (1)$$

where $y_{ij} = \{LoS_{ij}, c_{ij}\}$ indicates one of the two outcomes: long LoS (whether over 10 days), LoS_{ij}, or hospital costs, c_{ij}. The variables $HFRS_{ij}^L$, $HFRS_{ij}^I$ and $HFRS_{ij}^H$ indicate whether the patient was categorised as having low, intermediate or high frailty risk, with those having a zero frailty risk score forming the reference category. The regressions controlled for various patient characteristics, described above, indicated by vector X. u_j is a hospital fixed and ϵ_{ij} is a classical error term.

Our main interest was in the regression coefficients for δ_L, δ_I, and δ_H. If these were positive and significant, then those with low, intermediate, and high frailty risk had a higher probability of long LoS of stay or higher costs than patients assessed as having zero frailty risk.

We employed a logistic regression model to analyse long LoS and a generalised linear model (GLM) to analyse hospital costs. As a robustness check we also employed a Poisson model to analyse LoS. Analyses were conducted using Stata 15 (College Station, TX, USA). Forest plots were used to depict the results.

A.4 Full regression results

Table A2 reports the full regression results for the long LoS and hospital costs.

Table A2: Regression results of all population

**Variables**	**Long length of stay**<br>Odds ratio (95% CI)	**Hospital costs**<br>Coefficient (95% CI)
Zero risk(ref)		
Low risk	1.923***(1.792-2.063)	1926***(1655-2197)
Intermediate risk	2.713***(2.488-2.958)	4284***(3916-4652)
High risk	6.650***(3.064-14.433)	16613***(12827-20399)
Age (75-79) (ref)		
Age (80-84)	0.998(0.943-1.057)	55(-195-304)
Age (85+)	0.986(0.921-1.055)	349**(54-643)
Gender (female) (ref)		
Gender (male)	1.090***(1.035-1.149)	378***(150-605)
Secondary hospital (ref)		
Tertiary hospital	1.632***(1.535-1.734)	3310***(3037-3583)
Admission not through ED (ref)		
Admission through ED	0.936(0.860-1.020)	732***(350-1114)
CCI = 0 (ref)		
CCI = 1	0.912**(0.848-0.981)	882***(564-1202)
CCI = 2+	1.019(0.852-1.220)	786*(-49-1622)
Number of operations	1.279***(1.242-1.317)	4955***(4817-5093)
URBMI (ref)		
UEBMI	0.905***(0.844-0.971)	544***(238-849)
Reimbursement rate	4.975***(4.142-5.976)	2307***(1542-3072)
Number of patients	34731	34731
C-statistic / Adjusted R2	0.632	0.178

Notes: Significance levels: * p <0.1 ** p <0.05 *** p <0.001.

A.5 Analysis of length of stay with Poisson regression

As a robustness check for length of stay we also performed a Poisson regression. The unconditional means for the whole sample and by frailty risk category are reported in Table A3 with the regression results are shown in Table A4.

The mean LoS is 7.1 days for someone classified as zero frailty risk. Conditional on other characteristics, those in progressively higher risk categories are expected to have significantly longer LoS. For example, somebody with high frailty risk is expected to stay 7.5 days longer than someone with zero frailty risk. This is consistent with the results of the logistic regression examining the probability of long LoS.

Table A3: Description of length of stay

Variables	All samples ($n = 34,731$)		Zero risk ($n = 7715, 22.21\%$)		Low risk ($n = 21,667, 62.39\%$)		Intermediate risk ($n = 5,320, 15.32\%$)		High risk ($n = 29, 0.08\%$)	
	Mean	SE	Mean	SE	Mean	SE	Mean	SE	Mean	SE
Length of stay	8.7	0.04	7.1	0.07	8.7	0.04	10.7	0.17	19.8	4.76

Table A4: Poisson regression results for length of stay

Variables	Margin	P value	95% CI
HFRS = 0 (ref)			
Low risk	1.924***	<0.001	1.841, 2.007
Intermediate risk	3.361 ***	<0.001	3.256, 3.465
High risk	7.499 ***	<0.001	6.782, 8.217
Age (75-79) (ref)			
Age (80-84)	-0.108 ***	0.003	-0.179, -0.037
Age (85+)	-0.064	0.135	-0.147, 0.020
Female (ref)			
Male	0.181 ***	<0.001	0.117, 0.246
Secondary Hospital (ref)			
Tertiary Hospital	1.010 ***	<0.001	0.934, 1.087
Admission not through ED (ref)			
Admission through ED	-0.419***	<0.001	-0.52, -0.312
CCI = 0 (ref)			
CCI = 1	-0.125 ***	0.006	-0.215, -0.035
CCI = 2+	0.602 ***	<0.001	0.385, 0.820
No. of operations	0.936 ***	<0.001	0.905, 0.967
URBMI (ref)			
UEBMI	-0.039	0.370	-0.125, 0.046
Reimbursement rate	5.176 ***	<0.001	4.953, 5.400
Number of patients	34731		
Prob >chi2	<0.001		

Notes: Significance levels: * p <0.1 ** p <0.05 *** p <0.001.

A.6 Regression results of subgroup population

We ran the regressions for sub-groups of the analytical sample. For each sub-group Figure A1 and Table A5 report the results pertaining to the frailty risk groups, with coefficients for the other explanatory variables omitted. For all sub-groups, we find that the probabilities of a long LoS and that hospital costs increase monotonically across frailty risk categories.

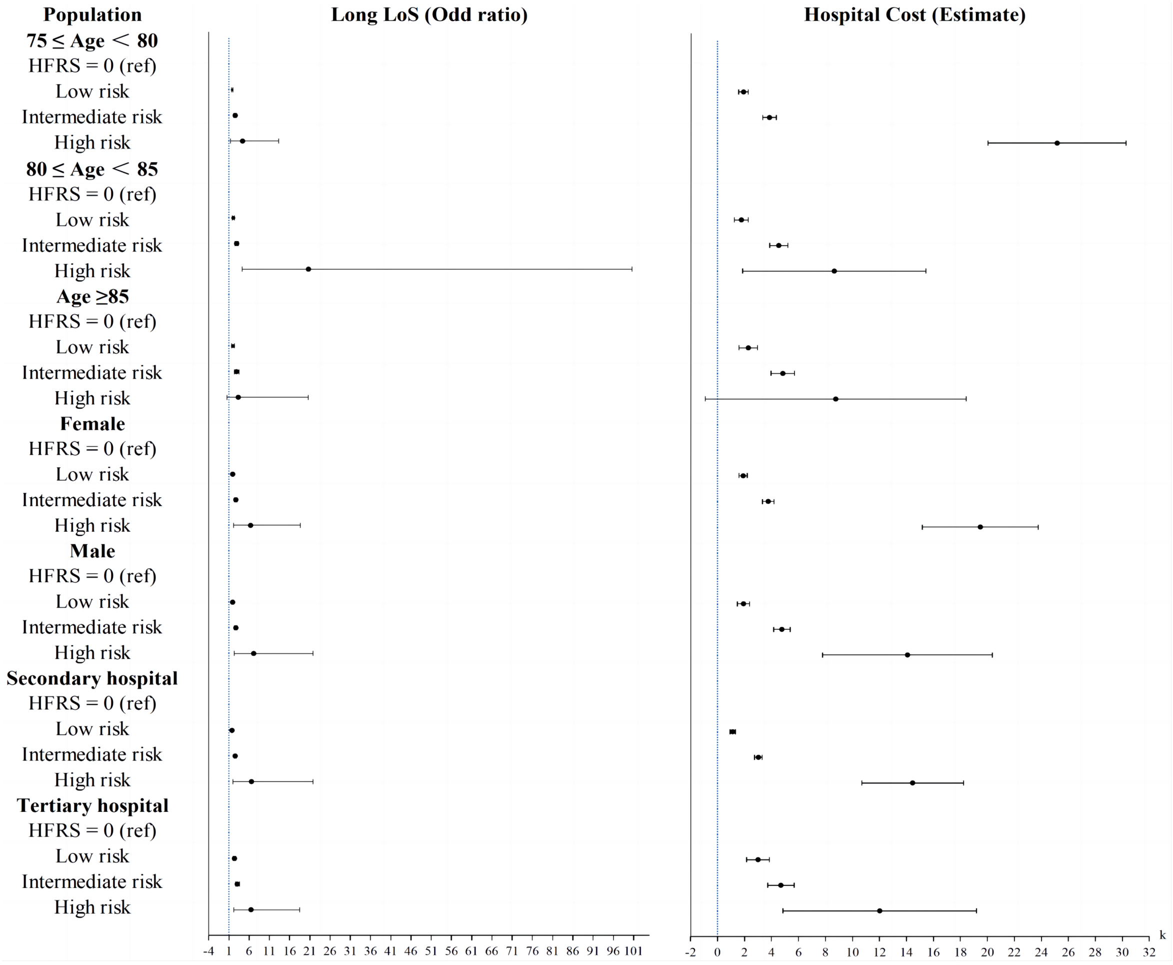

Figure A1: Forest plot of regression results for HFRS categories for the sub-groups (other coefficients suppressed)

Table A5: Regression results of subgroup population

**Subgroup population**	**Long length of stay Odd ratio (95% CI)**	**Hospital costs Estimate (95% CI)**
75 $\leq$ **Age** < 80 (N = 17,107)		
Low risk	1.813***(1.647-1.996)	1932***(1579-2285)
Intermediate risk	2.557***(2.258-2.896)	3852***(3343-4362)
High risk	4.377***(1.437-13.331)	25172***(20054-30290)
80 $\leq$ **Age** < 85 (N = 10,953)		
Low risk	2.089***(1.835-2.379)	1770***(1254-2286)
Intermediate risk	2.916***(2.497-3.404)	4538***(3859-5216)
High risk	20.66***(4.242-100.672)	8654**(1868-15439)
**Age** $\geq$ 85 (N = 6,671)		
Low risk	2.010***(1.691-2.389)	2284***(1594-2973)
Intermediate risk	2.878***(2.358-3.513)	4841***(3973-5709)
High risk	3.315(0.535-20.553)	8762*(-897-18422)
**Female** (N = 17,499)		
Low risk	1.932***(1.747-2.137)	1904***(1594-2215)
Intermediate risk	2.708***(2.394-3.064)	3759***(3338-4180)
High risk	6.365***(2.174-18.631)	19477***(15182-23773)
**Male** (N = 17,232)		
Low risk	1.915***(1.737-2.112)	1923***(1477-2369)
Intermediate risk	2.717***(2.407-3.067)	4766***(4158-5375)
High risk	7.124***(2.324-21.838)	14081***(7793-20370)
**Secondary hospital** (N = 24,912)		
Low risk	1.763***(1.627-1.911)	1124***(934-1313)
Intermediate risk	2.543***(2.285-2.829)	3032***(2751-3312)
High risk	6.573***(1.981-21.809)	14457***(10688-18225)
**Tertiary hospital** (N = 9,819)		
Low risk	2.369***(2.052-2.735)	3003***(2156-3850)
Intermediate risk	3.064***(2.614-3.591)	4694***(3709-5678)
High risk	6.462***(2.257-18.503)	12021***(4858-19184)

Notes: Significance levels: * p <0.1 ** p <0.05 *** p <0.001. Reference group: HFRS=0. For each subgroup the control variables are suppressed but are the same as the full population regression model (shown in Table A2).

References

[1] Charlson M, Pompei P, Ales K, MacKenzie C. A new method of classifying prognostic comorbidity in longitudinal studies: development and validation. J Chronic Dis. 1987;40(5):373-83.

[2] Quan H, Sundararajan V, Halfon P, Fong A, Burnand B, Luthi JC, et al. Coding Algorithms for Defining Comorbidities in ICD-9-CM and ICD-10 Administrative Data. Medical Care. 2005;43(11):1130-9.

[3] Bannay A, Chaignot C, Blotière P, Basson M, Weill A, Ricordeau P, et al. The Best Use of the Charlson Comorbidity Index With Electronic Health Care Database to Predict Mortality. Medical Care. 2016;54(2):188-94.

[4] Toson B, Harvey L, Close J. New ICD-10 version of the Multipurpose Australian Comorbidity Scoring System outperformed Charlson and Elixhauser comorbidities in an older population. Journal of Clinical Epidemiology. 2016;79:62-9.

[5] Gilbert T, Cordier Q, Polazzi S, Street A, Conroy S, Duclos A. Combining the hospital frailty risk score with the Charlson and Elixhauser multimorbidity indices to identify older patients at risk of poor outcomes in acute care. Medical Care. 2024;62(2):117-24.
